# An observational study investigating the feasibility of smart glasses for one-on-one nursing education during the COVID-19 pandemic in Taiwan

**DOI:** 10.1097/MD.0000000000048273

**Published:** 2026-04-03

**Authors:** Shiow-Jyu Tzou, Yi-Chen Wang, Hsiao-Mei Wang, Yu-Jyuan Wang, Chung-Bao Hsieh, Yung-Kuo Lee, Yu-Chiuan Wu, Po-Chun Lee, Tian-Huei Chu, Chou-Yuan Ko

**Affiliations:** a Medical Laboratory, Medical Education and Research Center, Kaohsiung Armed Forces General Hospital, Kaohsiung, Taiwan; b Institute of Medical Science and Technology, National Sun Yat-sen University, Kaohsiung, Taiwan; c Department of Internal Medicine, Division of Cardiology, Kaohsiung Armed Forces General Hospital, Kaohsiung, Taiwan; d Intensive Care Unit, Kaohsiung Armed Forces General Hospital, Kaohsiung, Taiwan; e Nursing Department, Kaohsiung Armed Forces General Hospital, Kaohsiung, Taiwan; f Department of Surgery, Kaohsiung Armed Forces General Hospital, Kaohsiung, Taiwan; g Department of Internal Medicine, Division of Gastroenterology and Hepatology, Kaohsiung Armed Forces General Hospital, Kaohsiung, Taiwan.

**Keywords:** familiarity, nursing education, satisfaction, smart glasses, students, teachers, the rating of smart glasses

## Abstract

During the Coronavirus disease 2019 (COVID-19) pandemic, smart technologies have been increasingly adopted in nursing education to reduce infection risk. However, the effectiveness and acceptance of smart glasses in clinical training remain unclear. This study aimed to identify factors influencing ratings of smart glasses and to examine rating correlations between paired nursing students and teachers following one-on-one training. We conducted a paired observational pilot study involving 30 nursing student–teacher pairs from Kaohsiung Armed Forces General Hospital, Taiwan, between November 2021 and December 2022. After completing clinical training using smart glasses, participants evaluated familiarity, satisfaction, time saving, and objectivity using a structured 5-point Likert-scale questionnaire. Group differences were analyzed using chi-square tests, independent *t*-tests, and 1-way ANOVA. Pearson correlation and linear regression analyses were performed to identify associated factors and inter-pair correlations. The results showed that teachers’ familiarity scores were significantly lower than students (*P* = .004). The higher time saving scores was significantly showed in teachers compared with students (*P* = .04). The satisfaction scores of students aged ≥24 years old were significantly lower than those of students aged <24 years old (*P* = .02). Teachers with ≥15 years of work experience had significantly lower familiarity scores than teachers with < 15 years of work experience (*P* = .03). Teachers with no experience using other smart technologies had significantly lower familiarity scores than teachers with experience using other smart technologies (*P* = .03). Among teachers, age (*P* = .01) and work experience (*P* = .04) were negatively correlated with familiarity scores. Finally, the familiarity (*P* = .03) and satisfaction scores (*P* = .02) of the paired students were positively correlated with the familiarity and satisfaction scores of the paired teachers. Smart glasses were less familiar to senior teachers and those without prior smart technology experience. Age and professional experience significantly influenced acceptance levels. Positive interdependence between paired students and teachers suggests that mutual adaptation may enhance smart technology implementation in clinical nursing education.

## 1. Introduction

The coronavirus disease 2019 (COVID-19) pandemic has profoundly disrupted health care systems worldwide, necessitating changes in clinical practice, communication, and education.^[1]^ To reduce transmission risk, many countries implemented infection control measures, including social distancing, restrictions on mass gatherings, and limitations on direct patient contact.^[2]^ In this context, health care professionals, particularly nurses, have faced increased occupational risks while continuing to provide essential care.^[3,4]^

Effective communication and coordination are fundamental to safe and efficient patient management.^[5]^ Over the past 2 decades, telemedicine technologies have been increasingly developed to facilitate remote consultation and clinical collaboration.^[6–8]^ The urgency of adopting such technologies became particularly evident during the COVID-19 pandemic, when minimizing physical contact while maintaining care quality became a priority.^[9]^

Smart glasses, wearable computing devices that enable real-time visual communication in a hands-free manner, have recently emerged as a promising tool in clinical practice and nursing education.^[10,11]^ These devices allow users to access textual and visual information within their field of view and support videoconferencing for consultation and supervision.^[9]^ Since their introduction into the medical market, several studies have explored the feasibility and applicability of smart glasses in clinical training and patient care settings.^[12–16]^ For example, the a German acronym for knowledge transfer care study demonstrated that mobile smart devices can help bridge tacit knowledge gaps and improve workflow efficiency in clinical units.^[17]^ Pilot studies have also reported positive perceptions of smart glasses in nursing training and task performance.^[18]^

However, most existing studies have primarily focused on feasibility and general user perceptions, with limited investigation into the specific determinants influencing user evaluations. To date, the impact of demographic and professional characteristics – such as age, work experience, clinical ladder, work department, and prior usage experience – on smart glasses ratings has not been systematically examined.

Understanding these influencing factors is essential for optimizing technology adoption strategies and improving training effectiveness in nursing education and clinical practice. Therefore, this study aimed to: assess user ratings of smart glasses in nursing education and clinical settings; examine the influence of demographic and professional characteristics on these ratings; and provide evidence to inform the implementation of smart wearable technologies in nursing practice and training.

## 2. Material and methods

### 2.1. Study design

This observational study was conducted in accordance with the Strengthening the Reporting of Observational Studies in Epidemiology (STROBE) guidelines. The study aimed to evaluate the feasibility of using smart glasses in one-on-one nursing education during the COVID-19 pandemic. Data collection was performed between November 3, 2021 and December 31, 2022 at Kaohsiung Armed Forces General Hospital (KAFGH), a regional teaching hospital in southern Taiwan. A one-to-one teaching model was implemented in which each nursing student was paired with an experienced nursing teacher during clinical training. After completion of the training session using smart glasses, both students and teachers completed a structured questionnaire evaluating their perceptions of the technology.

### 2.2. Participants

Participants were recruited from nursing staff and nursing students enrolled in a 2-year postgraduate nurse training program at KAFGH. Sample size estimation was performed using G*Power software to ensure adequate statistical power for detecting group differences.^[19]^ A total of 30 nursing students and 30 nursing teachers were included in the study. Because the teaching design was based on a one-to-one training format, each student was paired with 1 teacher, resulting in 30 matched student–teacher pairs. Inclusion criteria were: participation in the smart-glasses-assisted clinical training program, completion of the full training session, and willingness to complete the study questionnaire. Participants who did not complete the training session or declined to complete the questionnaire were excluded from the analysis.

### 2.3. Ethical approval

This study was reviewed and approved by the Institutional Review Board (IRB) of KAFGH (Approval number: KAFGHIRB 110-038). All procedures were conducted in accordance with the Declaration of Helsinki. Prior to participation, the study purpose and procedures were explained to all participants. Participation was voluntary, and participants were informed that they could withdraw from the study at any time without affecting their work or training. Written informed consent was obtained from all participants before data collection.

### 2.4. Smart glasses

**Figure 1.**
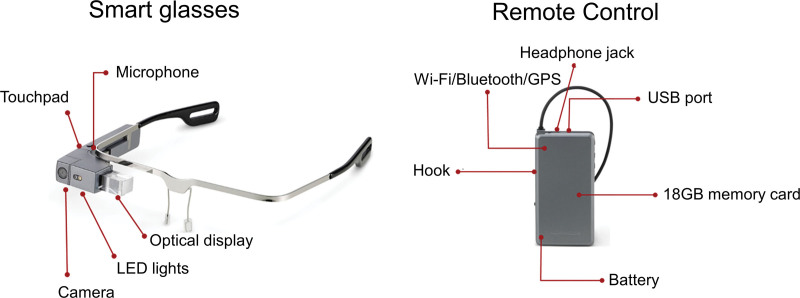
The description of the teaching/guidance system used in this study. The illustration of smart technology used in the present research, includes smart glasses (J-102) and remote control.

The smart-glasses-assisted teaching system used in this study was developed by Peer Giant System Inc. (New Taipei City, Taiwan). The system consisted of smart glasses (model J-102) and a remote control unit (Fig. 1). The smart glasses included a microphone, touchpad, optical display, light-emitting diode light, and front-facing camera, allowing real-time visual communication and hands-free operation. The remote control unit contained a Universal Serial Bus port, headphone jack, memory card (18 GB), battery module, Wi-Fi connectivity, Bluetooth, and Global Positioning System functions. The system was integrated into a Standard Operating Procedure medical teaching platform, which provided the following functions: hands-free operation through augmented reality display, standard operating procedure-based instructional guidance for clinical procedures, Video playback for training demonstrations, Self-assessment summary sheets for participants, and Real-time communication and remote collaboration. Using this system, teachers were able to observe students’ clinical procedures through the smart-glasses camera and provide immediate feedback or evaluation.

### 2.5. Data collection procedure

Following completion of the clinical training session, all participants completed a structured questionnaire designed to evaluate their perceptions of the smart-glasses system. The questionnaire collected demographic and professional information, including: age, gender, department, clinical ladder level, work experience, previous experience using other smart technologies, frequency of smart glasses use, and whether the system met their expectations. Participants also rated the smart-glasses system across 4 attributes: familiarity, satisfaction, time saving, and objectivity. Each attribute was evaluated using a 5-point Likert scale, where: 1 = very poor, 2 = poor, 3 = fair, 4 = good, and 5 = excellent.^[20]^

### 2.6. Statistical analyses

Differences in the distribution of each collected variable (gender, age, work experience, clinical ladder, department, and experience using other smart technologies) between student and teacher groups were analyzed using chi-square tests. The impact of each variable on whether expectations were met was also investigated within the student and teacher groups using chi-square tests. For the correlation analysis between different variables, this study used *Pearson* correlation analysis. All comparisons between groups were analyzed by 1-way ANOVA or 2-tailed Student *t* test. The results are presented as mean ± SD, and *P* < .05 was considered statistically significant. We used GraphPad Prism 7.0 (GraphPad Software) and SPSS Statistics 26 software (IBM Corp., Armonk) for the statistical calculations.

## 3. Results

### 3.1. Sample characteristics

A total of 30 student–teacher pairs (N = 60 participants) from a regional teaching hospital in southern Taiwan were enrolled during the COVID-19 pandemic. Among students, 13.3% (4/30) were male and 86.7% (26/30) were female (Table 1). 43.3% (13/30) of students were <24 years old (mean), and 56.7% (17/30) were ≥24 years old. In addition, 50% (15/30) of the students have worked at KAFGH for 1 and 2 years respectively. The clinical ladder of 90% (27/30) students was N, and the clinical ladder of 10% (3/27) students was N1. 33.3% (10/30) and 20% (6/30) of students were engaged in internal medicine and surgery respectively. The ratio of students worked at other wards is 46.7% (14/30). 63.3% (19/30) students have the experience of using other smart technology. Among teachers, 3.3% (1/30) are male and 96.7% (29/30) are female. 50% (15/30) of the teachers were <40 years old (average) and ≥40 years old respectively. Additionally, 60% (18/30) and 40% (12/30) of teachers had <15 years (mean) and ≥15 years of work experience at KAFGH respectively. The clinical ladders of 30% (9/30), 63.3% (19/30), and 6.7% (2/30) of teachers were N1, N2, and N3 respectively. Teachers engaged in internal medicine and surgery accounted for 36.7% (11/30) and 20% (6/30) respectively. The ratio of teachers worked at other wards is 43.3% (13/30). 60% (18/30) of teachers have experience in using other smart technology.

**Table 1:** The characteristics of students and teachers.

Variable	Number of participants (n = 60)	Students (n = 30)	Teachers (n = 30)	*P* value†
Gender				
Male	5	4 (13.3%)	1 (3.3%)	.35
Female	55	26 (86.7%)	29 (96.7%)	
Age (yr)				
<24 (student)	13	13 (43.3%)	0 (0%)	<.001*
≥24 (student)	17	17 (56.7%)	0 (0%)	
<40 (teacher)	15	0 (0%)	15 (50%)	
≥40 (teacher)	15	0 (0%)	15 (50%)	
Working experience at KAFGH (yr)				
1 (student)	15	15 (50%)	0 (0%)	<.001*
2 (student)	15	15 (50%)	0 (0%)	
<15 (teacher)	18	0 (0%)	18 (60%)	
≥15 (teacher)	12	0 (0%)	12 (40%)	
Clinical ladder				
N	27	27 (90%)	0 (0%)	<.001*
N1	12	3 (10%)	9 (30%)	
N2	19	0 (0%)	19 (63.3%)	
N3	2	0 (0%)	2 (6.7%)	
Department				
Internal medicine	21	10 (33.3%)	11 (36.7%)	>.99
Surgery	12	6 (20%)	6 (20%)	
Other	27	14 (46.7%)	13 (43.3%)	
Previous experience of smart technology use				
Yes	37	19 (63.3%)	18 (60%)	0.79
No	23	11 (36.7%)	12 (40%)	

† Chi-square test.

* *P* < .001.

### 3.2. Overall ratings of smart glasses

**Figure 2.**
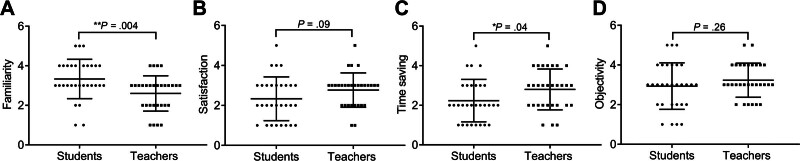
The ratings of smart glasses between students and teachers. (A) Familiarity, (B) satisfaction, (C) time saving, and (D) objectivity scores in the student and teacher groups. Data were mean ± SD (*P < .05, **P < .01). SD = standard deviation.

Attributes of the checklist used in this study included familiarity, satisfaction, time saving, and objectivity. Attribute ratings were based on a 5-point Likert scale: 1 (very poor), 2 (poor), 3 (fair), 4 (good) and 5 (excellent).^[20]^ Among the student population, the mode scores for familiarity, satisfaction, time saving, and objectivity were 4 (10/30, 33.3%), 2/3 (9/30, 30%), 2 (9/30, 30%), and 3 (11/30, 36.7%), respectively (Table 2). Additionally, the teacher group received a score of 3 on all attributes including familiarity (14/30, 46.7%), satisfaction (16/30, 53.3%), time saving (10/30, 33.3%), and objectivity (13/30, 43.3%; Table 3), suggesting a generally moderate evaluation of smart glasses among the teaching staff. Teachers had significantly lower familiarity scores (2.60 ± 0.89, n = 30) compared to students (3.33 ± 0.99, n = 30; **P = .004; Fig. 2), and this finding suggests that teachers may be less accustomed to the use of newly introduced smart technologies compared with nursing trainees. Interestingly, the higher time saving scores was significantly showed in teachers (3.00 ± 1.11, n = 30) compared with students (2.43 ± 1.25, n = 30; *P = .04), indicating that experienced nursing staff may perceive greater efficiency benefits when using smart glasses during clinical instruction. Other attributes showed no significant differences between teachers and students.

**Table 2:** Checklist scores of smart glasses in the students (n = 30).

Attributes	Checklist scores*					Mode score
	1 (very-poor)	2 (poor)	3 (fair)	4 (good)	5 (excellent)	
Familiarity	2 (6.7%)	2 (6.7%)	13 (43.3%)	10 (33.3%)	3 (10%)	4
Satisfaction	8 (26.7%)	9 (30%)	9 (30%)	3 (10%)	1 (3.3%)	2, 3
Time saving	8 (26.7%)	9 (30%)	6 (20%)	3 (10%)	1 (3.3%)	2
Objectivity	4 (13.3%)	6 (20%)	11 (36.7%)	6 (20%)	3 (10%)	3

* Attributes scored on a 5-point Likert scale.

**Table 3:** Checklist scores of smart glasses in the teachers (n = 30).

Attributes	Checklist scores*					Mode score
	1 (very-poor)	2 (poor)	3 (fair)	4 (good)	5 (excellent)	
Familiarity	4 (13.3%)	8 (26.7%)	14 (46.7%)	4 (13.3%)	0 (0%)	3
Satisfaction	2 (6.7%)	8 (26.7%)	16 (53.3%)	3 (10%)	1 (3.3%)	3
Time saving	3 (10%)	9 (30%)	10 (33.3%)	7 (23.3%)	1 (3.3%)	3
Objectivity	0 (0%)	6 (20%)	13 (43.3%)	9 (30%)	2 (6.7%)	3

* Attributes scored on a 5-point Likert scale.

### 3.3. Factors associated with smart glasses ratings

#### 3.3.1. Age

**Figure 3.**
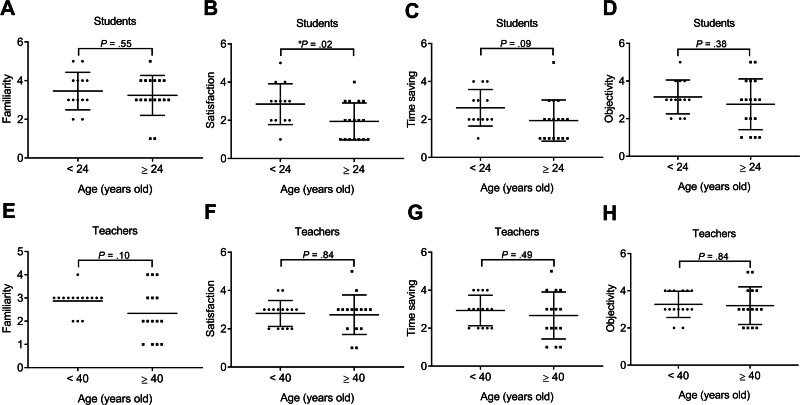
The rating of smart glasses in the students and teachers of different ages. (A) Familiarity, (B) satisfaction, (C) time saving, and (D) objectivity scores in the students with the ages of <24 or ≥24 years old. (E) Familiarity, (F) satisfaction, (G) time saving, and (H) objectivity scores in the teachers with the ages of <40 or ≥40 years old. Data were mean ± SD (*P < .05). SD = standard deviation.

**Figure 4.**
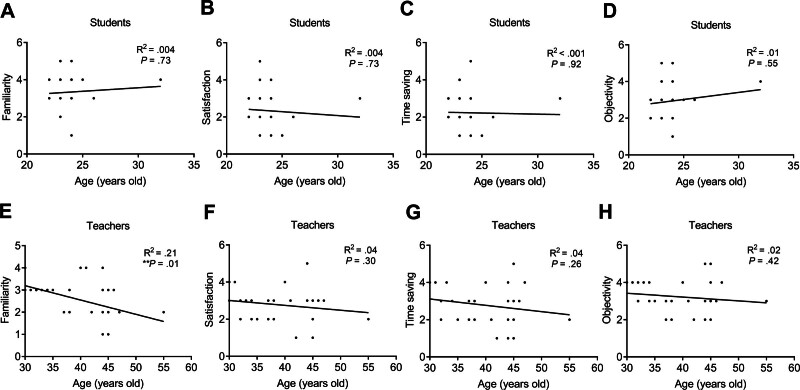
The correlation between the rating of smart glasses and ages in student and teacher groups. The *Pearson* analysis for the correlation between the ages and (A) familiarity, (B) satisfaction, (C) time saving, or (D) objectivity scores in the students. The *Pearson* analysis for the correlation between the ages and (E) familiarity, (F) satisfaction, (G) time saving, or (H) objectivity scores in the teachers (**P < .01).

Students aged ≥24 years (on average) had significantly lower satisfaction scores (1.94 ± 0.97, n = 17) compared to students aged <24 years (2.85 ± 1.07, n = 13; *P = .02; Fig. 3). No significant difference was shown in other attributes between <24-year-old and ≥24-year-old students. The checklist scores of all attributes between <40-year-old and ≥40-year-old teachers did not show a significant difference. The *Pearson* correlation analysis was used to study the relevance between age and checklist scores of all attributes in the students and teachers. In the students, age was not significantly correlated with the familiarity, satisfaction, time saving, and objectivity scores of checklists (Fig. 4). In the teacher population, age was significantly and negatively correlated with familiarity score (*R*^2^ = .21, *P = .01, n = 30), suggesting that older teachers tended to report lower familiarity with smart glasses technology. Moreover, the age was not significantly correlated with other attributes.

#### 3.3.2. Work experience

**Figure 5.**
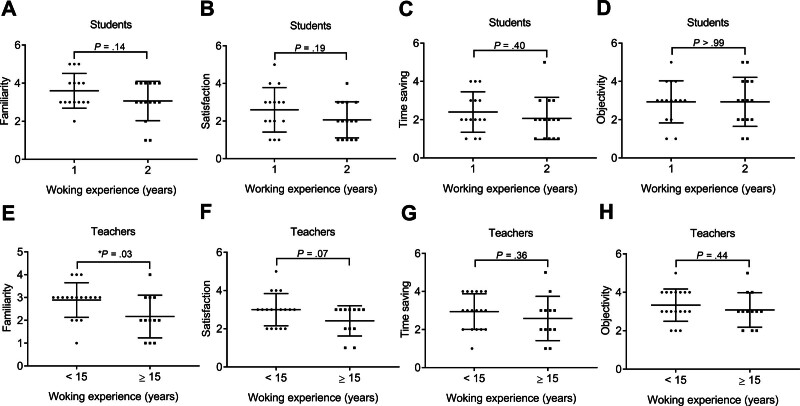
The rating of smart glasses in the students and teachers with different working experience. (A) Familiarity, (B) satisfaction, (C) time saving, and (D) objectivity scores in the students with 1-year or 2-year working experience. (E) Familiarity, (F) satisfaction, (G) time saving, and (H) objectivity scores in the teachers with a working experience of <15 or ≥15 years. Data were mean ± SD (*P < .05). SD = standard deviation.

**Figure 6.**
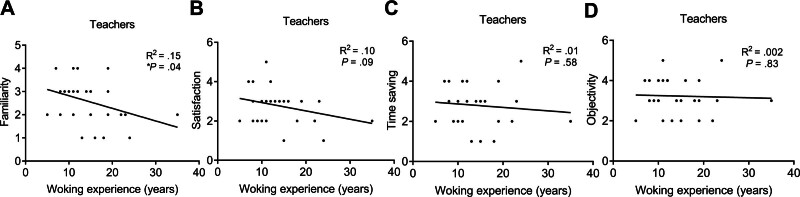
The correlation between the rating of smart glasses and working experience (years) in the teachers. The *Pearson* analysis for the correlation between the working experience (years) and (A) familiarity, (B) satisfaction, (C) time saving, or (D) objectivity scores in the teachers (*P < .05).

No significant difference was shown in the score of all attributes between students with 1- and 2-year work experience. Moreover, the familiarity score was significantly lower in the teachers with ≥15-year (mean) work experience (2.17 ± 0.94, n = 12) compared with <15-year work experience (2.89 ± 0.76, n = 18; *P = .03; Fig. 5). No significant difference was shown in the checklist score of other attributes between teachers with different work experiences. The correlation between work experience and checklist scores in the teachers was further investigated using the *Pearson* correlation analysis. It was shown that the familiarity score was significantly and negatively correlated with work experience (*R*^2^ = .15, *P = .04, n = 30; Fig. 6), indicating that longer professional experience was associated with lower familiarity with smart glasses technology. The correlation between other attribute scores and work experience has no statistical significance in the teachers.

#### 3.3.3. Experience using other smart technologies

**Figure 7.**
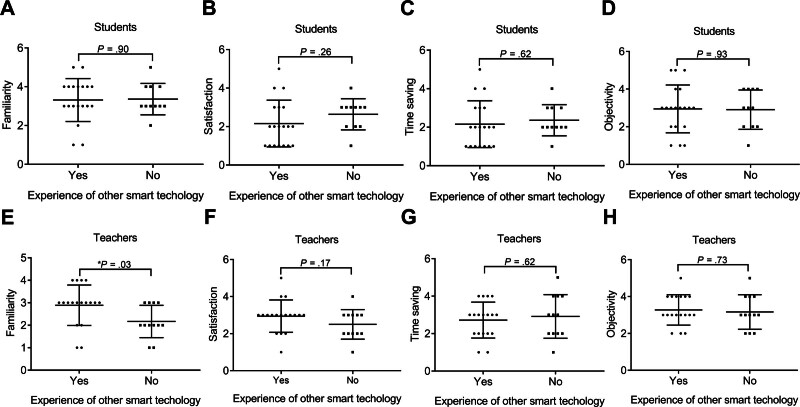
The rating of smart glasses in the students and teachers with/without the experience of using other smart technology. (A) Familiarity, (B) satisfaction, (C) time saving, and (D) objectivity scores in the students with or without the experience of using other smart technology. (E) Familiarity, (F) satisfaction, (G) time saving, and (H) objectivity scores in the teachers with or without the experience of using other smart technology. Data were mean ± SD (*P < .05). SD = standard deviation.

In the student population, the experience of using other smart technology did not impact the checklist score of all attributes. Interestingly, the teachers with no experience using other smart technologies had significantly lower familiarity scores (2.17 ± 0.72, n = 12) than teachers with experience using other smart technologies (2.89 ± 0.90, n = 18; *P = .03; Fig. 7). The experience of using other smart technology did not affect the checklist score of other attributes in the teachers.

### 3.4. Correlation between paired students and teachers

**Figure 8.**
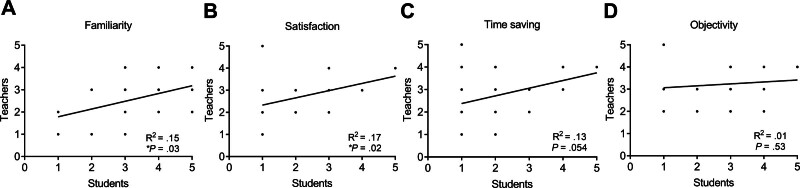
The correlation in the rating of smart glasses between students and teachers. The *Pearson* analysis for the correlation in (A) familiarity, (B) satisfaction, (C) time saving, or (D) objectivity scores between the student and teacher groups (*P < .05).

In this study, the paired students and teachers were enrolled. This study also investigated whether paired students and teachers influence each other’s checklist scores. Thus, the correlation of checklist scores between paired students and teachers will be analyzed using *Pearson* correlation analysis. Interestingly, the familiarity (*R*^2^ = .15, *P = .03, n = 30) and satisfaction (*R*^2^ = .17, *P = .02, n = 30) scores between paired students and teachers have a significantly positive correlation (Fig. 8). These findings suggest that the perceptions of smart glasses among students and teachers may influence each other during the learning process.

## 4. Discussion

This study investigated the feasibility of using smart glasses for one-on-one nursing education during the COVID-19 pandemic. The main findings were as follows: teachers demonstrated significantly lower familiarity scores than students, older students reported lower satisfaction scores compared with younger students, teachers with longer work experience showed significantly lower familiarity with smart glasses, and familiarity and satisfaction scores were positively correlated between paired students and teachers. These findings provide insight into factors influencing the acceptance of smart glasses in nursing education settings.

In this study, teachers demonstrated significantly lower familiarity scores than students. One possible explanation is the difference in technology adoption between younger and older users. Previous studies have suggested that younger individuals tend to adopt and adapt to emerging digital technologies more easily than older users.^[21,22]^ A previous study indicated that the frequency of using new technology is relatively higher in the younger than the older.^[22]^ The high frequency of new technology use may cause indifference to smart glasses in the students, and this was further supported by our finding that an increased trend was shown in the use times of smart glasses in the students compared with teachers (*P* = .06; Fig. S1, Supplemental Digital Content, https://links.lww.com/MD/R617). In nursing education, younger learners are often more accustomed to using digital tools and mobile technologies in their learning environment, whereas experienced nurses may have relied more heavily on traditional teaching methods throughout their careers. As a result, teachers may require additional training and support to become familiar with new educational technologies such as smart glasses.

Age-related differences were also observed in students, as older students reported significantly lower satisfaction scores than younger students. Age has been reported to influence the ability to acquire new technological skills and adapt to digital tools.^[21]^ Although the age difference among students in this study was relatively small, the results suggest that even modest differences in age may influence perceptions of technology-based learning tools. Therefore, tailored training or orientation sessions may help improve user satisfaction when implementing smart technologies in nursing education.

Work experience was another important factor affecting familiarity with smart glasses among teachers. In the present study, teachers with ≥15 years of work experience demonstrated significantly lower familiarity scores, and work experience was negatively correlated with familiarity. This finding may reflect differences in prior exposure to digital technologies. Nurses with longer clinical careers may have received most of their professional training before the widespread adoption of smart technologies in healthcare education. Consequently, they may have had fewer opportunities to develop digital competencies compared with younger healthcare professionals.

In addition, teachers without prior experience using other smart technologies also showed lower familiarity scores. Previous research has shown that experience with digital tools can significantly influence users’ acceptance and perceived usability of new technologies.^[23-26]^ Individuals who are already familiar with digital or wearable devices may adapt more easily to smart glasses. These findings suggest that previous experience with related technologies may play an important role in facilitating the adoption of smart glasses in clinical education settings.

Another important finding of this study was the positive correlation between familiarity and satisfaction scores between paired students and teachers. This result suggests that teacher–student interaction may influence the learning experience when using smart technologies. Previous studies have indicated that effective interaction between teachers and students can significantly influence learning engagement and educational outcomes.^[27]^ When teachers demonstrate confidence and familiarity with educational tools, students may also feel more comfortable and engaged in the learning process. Therefore, improving educators’ familiarity with smart technologies may indirectly enhance students’ learning experiences.

The effect of a clinical ladder and working department on attribute scoring was also investigated in the students and teachers. Every attribute scoring was not significantly different in the students with different clinical ladders (Fig. S2, Supplemental Digital Content, https://links.lww.com/MD/R617), and similar results were shown in the teachers. Furthermore, every attribute scoring was not significantly different in the students working in different wards (Fig. S3, Supplemental Digital Content, https://links.lww.com/MD/R617), and similar results were also showed in the teacher population. In addition, we found that the use time of smart glasses was not significantly correlated with the attribute scores in the students and teachers (Fig. S4, Supplemental Digital Content, https://links.lww.com/MD/R617), and these findings further supported that age acts as a key factor for attribute scoring in the nursing the students and teachers. Finally, we studied which variable affected the expectation of smart glasses in the students (Table S1, Supplemental Digital Content, https://links.lww.com/MD/R618) and teachers (Table S2, Supplemental Digital Content, https://links.lww.com/MD/R618), and we found that gender, age, working experience, clinical ladder, working wards, and previous experiment using other smart technology did not significantly affect the expectation of smart glasses in the participants. A previous study reported that smart glasses can serve as a complement to routines and existing, but it can’t totally replace human presence in intensive care,^[28]^ and this may partially support the above findings in this research.

Smart glasses have increasingly been explored in healthcare education and clinical practice due to their ability to provide hands-free communication, real-time visual guidance, and remote collaboration.^[10,23-26]^ Previous studies have suggested that wearable technologies can enhance clinical training by enabling real-time feedback and improving communication between instructors and trainees.^[20]^ In the context of the COVID-19 pandemic, these technologies may also reduce unnecessary physical contact while maintaining effective educational interactions. Our findings further support the potential value of smart glasses as a complementary tool for nursing education. Despite these promising findings, several limitations should be acknowledged. First, the sample size was relatively small, consisting of only 30 pairs of students and teachers, which may limit the statistical power of the analyses. Second, this study was conducted in a single regional teaching hospital in Taiwan, which may limit the generalizability of the findings to other institutions or healthcare systems. Third, the results were based on self-reported questionnaire responses, which may introduce response bias. Finally, the cross-sectional design of the study does not allow causal relationships to be established between the examined factors and participants’ perceptions of smart glasses. Future studies with larger multicenter samples and longitudinal designs are needed to further evaluate the effectiveness of smart glasses in nursing education.

However, our findings are consistent with previous international studies examining the use of wearable technologies in healthcare education. A scoping review reported that smart glasses have been increasingly used in nursing education and can enhance student engagement and learning motivation, although technical limitations remain a challenge.^[29]^ In addition, a systematic review of Google Glass applications in medical settings demonstrated high feasibility and acceptability, particularly in clinical training and educational environments.^[11]^ Other studies have shown that smart glasses can facilitate remote supervision and communication in healthcare through real-time video sharing and telemedicine applications.^[9]^ Furthermore, research on nursing skill training indicated that smart glasses can improve perceived competency and support hands-free learning during clinical practice.^[30]^ Recent simulation-based nursing education studies also reported higher satisfaction and improved critical thinking among students using smart glasses.^[31]^

## 5. Limitations

Our study had several limitations. Firstly, it was limited by the small sample size of 30 paired nursing students and teachers. However, we should try to recruit >100 pairs of the participants in further research. It was also limited by the single-center study in the present research, and the multicenter research should be further performed in the future.

## 6. Conclusions

This pilot study identified several factors influencing the adoption of smart glasses in nursing education during the COVID-19 pandemic. Teachers demonstrated lower familiarity with smart glasses compared with students, and familiarity was negatively associated with age and work experience. In addition, prior experience with other smart technologies was associated with higher familiarity among teachers. Notably, familiarity and satisfaction scores were positively correlated between paired students and teachers, suggesting that educator–learner interaction may influence the acceptance of wearable technologies in clinical training. These findings highlight the importance of targeted training and technological support for educators when implementing smart technologies in nursing education. Further multicenter studies with larger sample sizes are needed to confirm these findings and explore the broader educational impact of wearable technologies.

## Acknowledgments

The authors thank Yi-Ting Wu, Yun-Yu Lin, and Meng-Hsun Wu (Laboratory of Research and Medical Education and Research Center, Kaohsiung Armed Forces General Hospital) for their statistics support.

## Author contributions

**Conceptualization:** Tian-Huei Chu, Chou-Yuan Ko.

**Data curation:** Shiow-Jyu Tzou, Yi-Chen Wang, Hsiao-Mei Wang, Yu-Jyuan Wang, Chung-Bao Hsieh, Yung-Kuo Lee.

**Formal analysis:** Shiow-Jyu Tzou, Yi-Chen Wang, Hsiao-Mei Wang, Yu-Jyuan Wang, Chung-Bao Hsieh, Yung-Kuo Lee.

**Investigation:** Shiow-Jyu Tzou, Yi-Chen Wang, Hsiao-Mei Wang, Yu-Jyuan Wang, Chung-Bao Hsieh, Yung-Kuo Lee.

**Resources:** Yu-Chiuan Wu, Po-Chun Lee.

**Supervision:** Tian-Huei Chu, Chou-Yuan Ko.

**Writing – original draft:** Shiow-Jyu Tzou, Yi-Chen Wang.

**Writing – review & editing:** Tian-Huei Chu, Chou-Yuan Ko.

## Supplementary Material
