# Supplementary material for: An observational study investigating the feasibility of smart glasses for one-on-one nursing education during the COVID-19 pandemic in Taiwan
Source: Medicine (Baltimore). 2026 Apr 3;105(14):e48273. doi: 10.1097/MD.0000000000048273 (PMC13052966; doi:10.1097/MD.0000000000048273)
Supplement: Supplementary file 1 [file medi-105-e48273-s001.pdf]

Supplementary Figure S1

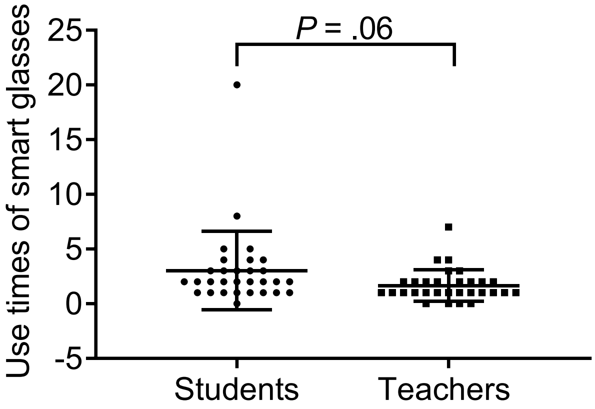

Supplementary Figure S1. The difference of using experience between students and teachers. The use times of smart glasses in students and teachers.

Supplementary Figure S2

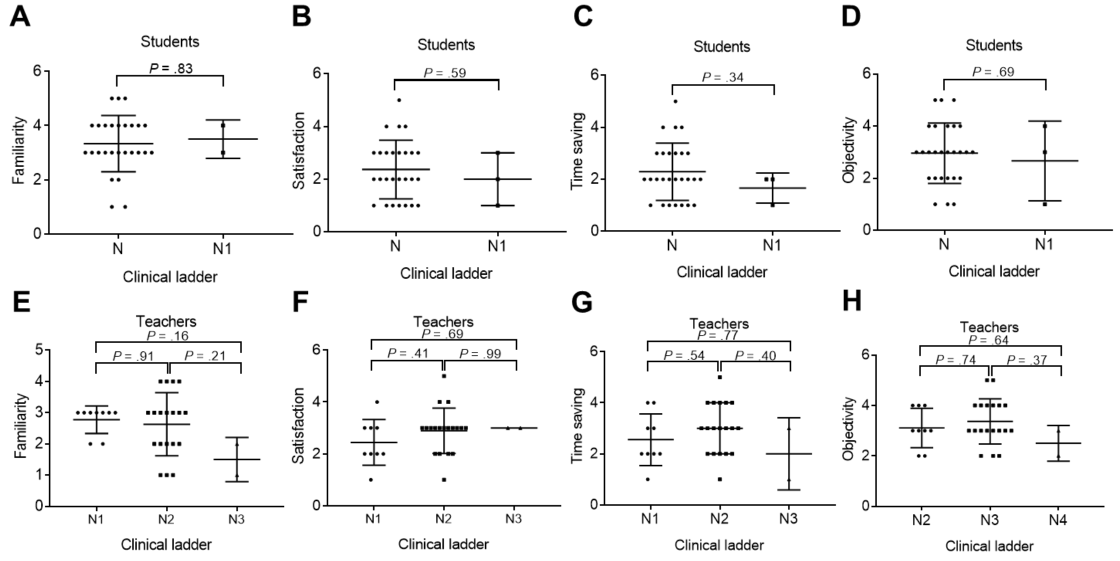

Supplementary Figure S2. The rating of smart glasses in the students and teachers with different clinical ladders. (A) Familiarity, (B) satisfaction, (C) time saving and (D) objectivity scores in the students with N or N1 levels. (E) Familiarity, (F) satisfaction, (G) time saving, and (H) objectivity scores in the teachers with N1 to N3 levels.

Supplementary Figure S3

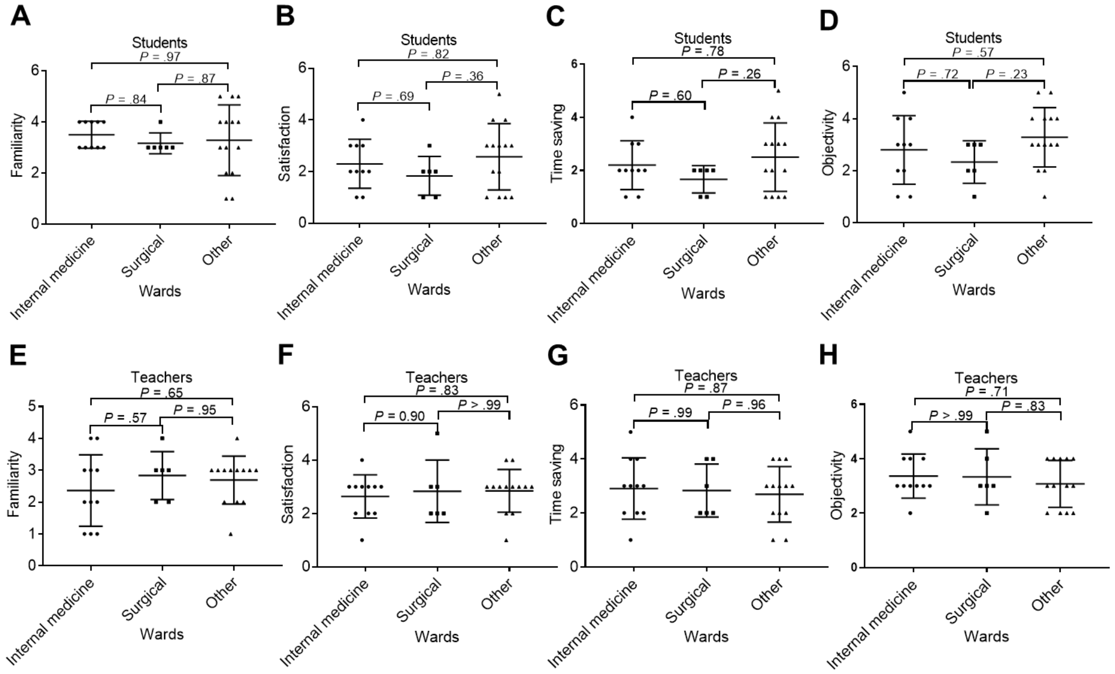

Supplementary Figure S3. The rating of smart glasses in the students and teachers worked at different departments. (A) Familiarity, (B) satisfaction, (C) time saving and (D) objectivity scores in the students worked at different wards. (E) Familiarity, (F) satisfaction, (G) time saving, and (H) objectivity scores in the teachers worked at different wards.

Supplementary Figure S4

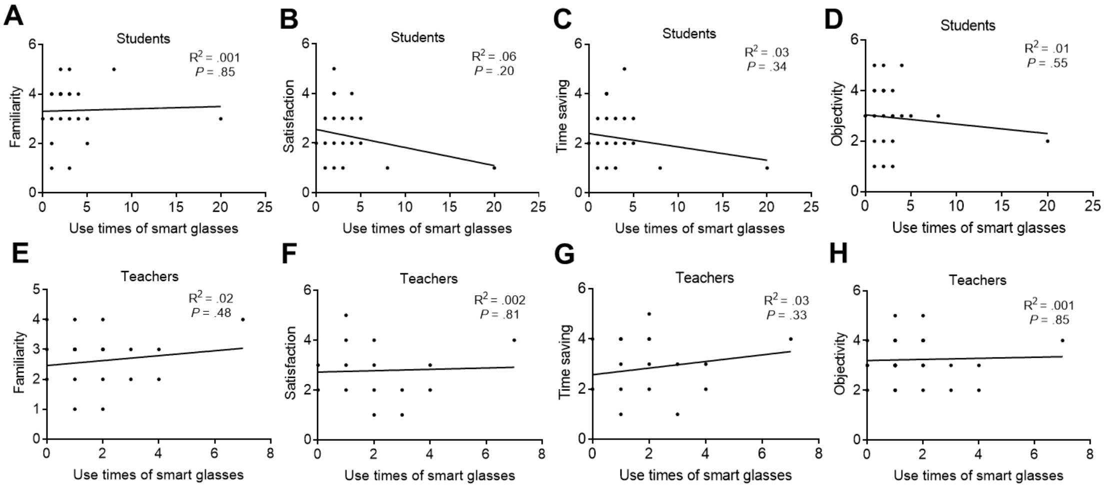

Supplementary Figure S4. The correlation between the rating of smart glasses and the using experience for smart glasses in the students and teachers. The *Pearson* analysis for the correlation between the use times of smart glasses and (A) familiarity, (B) satisfaction, (C) time saving, or (D) objectivity scores in the students. The *Pearson* analysis for the correlation between the use times of smart glasses and (E) familiarity, (F) satisfaction, (G) time saving, or (H) objectivity scores in the teachers.
