# Supplementary material for: An observational study investigating the feasibility of smart glasses for one-on-one nursing education during the COVID-19 pandemic in Taiwan
Source: Medicine (Baltimore). 2026 Apr 3;105(14):e48273. doi: 10.1097/MD.0000000000048273 (PMC13052966; doi:10.1097/MD.0000000000048273)
Supplement: Supplementary file 2 [file medi-105-e48273-s002.pdf]

Supplementary Table S1. The expectations for smart glasses in the students.

Variable	No. of students (n=30)	Whether meet expectations		*p* value[a]
		Yes (n=5)	No (n=25)	
Gender				
Male	4	1 (20%)	3 (12%)	.54
Female	26	4 (80%)	22 (88%)	
Age (years)				
< 24	13	3 (60%)	10 (40%)	.16
≥ 24	17	2 (40%)	15 (60%)	
Working years				
1	15	2 (40%)	13 (52%)	> .99
2	15	3 (60%)	12 (48%)	
Clinical ladder				
N	27	5 (100%)	22 (88%)	> .99
N1	3	0 (0%)	3 (12%)	
Department				
Internal medicine	10	2 (40%)	8 (32%)	.67
Surgery	6	0 (0%)	6 (24%)	
Other	14	3 (60%)	11 (44%)	
Previous experience of smart technology use				
Yes	19	5 (100%)	14 (56%)	.13
No	11	0 (0%)	11 (44%)	

[a]*Chi-square* test

Supplementary Table S2. The expectations for smart glasses in the teachers.

Variable	No. of teachers (n=30)	Whether meet expectations		*p* value[a]
		Yes (n=10)	No (n=20)	
Gender				
Male	1	0 (0%)	1 (4%)	> .99
Female	29	100 (100%)	19 (96%)	
Age (years)				
< 40	15	5 (50%)	10 (50%)	> .99
≥ 40	15	5 (50%)	10 (50%)	
Working years				
< 15 (Teacher)	18	8 (80%)	10 (50%)	.23
≥ 15 (Teacher)	12	2 (20%)	10 (50%)	
Clinical ladder				
N1	9	3 (30%)	6 (30%)	.85
N2	19	7 (70%)	12 (60%)	
N3	2	0 (0%)	2 (10%)	
Department				
Internal medicine	11	3 (30%)	8 (40%)	.69
Surgery	6	3 (30%)	3 (15%)	
Other	13	4 (40%)	9 (45%)	
Previous experience of smart technology use				
Yes	18	7 (70%)	11 (55%)	.69
No	12	3 (30%)	9 (45%)	

[a]*Chi-square* test
